# Bridging perception and reality: understanding Chinese women’s behavioral intentions toward robot-assisted gynecologic surgery

**DOI:** 10.3389/fpubh.2026.1743010

**Published:** 2026-04-02

**Authors:** Zhengzijin Yang, Siyu Zhao

**Affiliations:** 1 School of Information Management, Central China Normal University, Wuhan, China; 2 Key Laboratory of Aerospace Information Security and Trusted Computing, School of Cyber Science and Engineer, Wuhan University, Wuhan, China

**Keywords:** behavioral intention, laparoscopic hysterectomy, radical hysterectomy, robotic gynecologic surgery, robotic surgery

## Abstract

**Background:**

Robot-assisted gynecological surgery (RAGS) represents a major advancement in minimally invasive care with demonstrated clinical benefits. Yet, its adoption depends on individual acceptance shaped by complex psychosocial factors, particularly salient in China due to healthcare disparities and information asymmetry. Prior studies on health technology acceptance have seldom examined discrepancies between individual expectations and actual clinical outcomes. This study investigates Chinese women’s acceptance of RAGS, introducing the concept of perceived postoperative difference to capture the perception–reality gap and its influence on behavioral intention.

**Methods:**

A cross-sectional survey was conducted among Chinese women using convenience sampling, yielding 546 valid responses. An extended UTAUT framework incorporating trust, perceived risk, and perceived postoperative difference (PPD) was applied. The perception–reality gap was quantified by matching participants’ perceived postoperative outcomes with objective clinical data. Structural Equation Modeling (SEM) tested the framework, and multi-group SEM examined behavioral differences across gap-defined groups. Analysis of variance and chi-square tests were used to assess demographic variations among these groups.

**Results:**

The model showed good fit. Analyses indicated that effort expectancy was the strongest predictor of behavioral intention, followed by perceived postoperative difference and performance expectancy. Facilitating conditions enhanced both behavioral intention and performance expectancy, while trust increased both and reduced perceived risk. Multi-group analysis revealed notable heterogeneity. In the overestimation group, behavioral intention mainly depended on effort expectancy, while social influence and perceived risk reduced intention. In the alignment group, facilitating conditions and trust improved performance expectancy. In the underestimation group, behavioral intention was primarily shaped by performance expectancy and social influence. Higher education and technical jobs characterized the overestimation group, whereas lower education and unstable employment defined the underestimation group.

**Conclusion:**

This study reveals that the perception-reality gap reconfigures women’s decision pathways, demonstrating that adoption mechanisms are highly heterogeneous across perceptual groups. These gaps reflect underlying socioeconomic and structural factors, highlighting the necessity of targeted patient education and communication strategies. This research expands the boundaries of traditional technology adoption models in high-risk medical contexts, refines user acceptance theory, and provides valuable empirical evidence for formulating personalized communication strategies and optimizing the promotion of novel medical technologies.

## 1 Introduction

The development of surgical practice has consistently been driven by the pursuit of minimally invasive and greater precision. In 2005, the U.S. Food and Drug Administration (FDA) approved robotic-assisted surgery for gynecologic procedures. Since then, robotic-assisted gynecologic surgery (RAGS) has advanced rapidly (1). Compared with open surgery and conventional laparoscopic surgery (LS), robotic platforms significantly enhance surgical precision and safety through high-definition three-dimensional (3D) visualization, physiological tremor filtration, and articulated instruments with superior dexterity. These features improve stability and control in complex gynecologic procedures (2). Existing studies have suggested that robotic surgery (RS) offers several potential advantages, including reduced intraoperative blood loss, fewer complications, shorter hospital stays, and faster postoperative recovery (3, 4). However, because of differences in case composition and healthcare systems, some studies reported comparable clinical outcomes between robotic and conventional laparoscopic surgery, whereas others raised concerns about longer operative times and higher costs (5–7). When objective evidence remains inconclusion, public interpretation relies on pre-existing cognitive lenses. For most women without medical training, the complexity of specialized clinical data shifts risk–benefit assessments toward subjective perception. Consequently, as medical information becomes more complex and clinical outcomes more inconsistent, subjective perception becomes increasingly influential in individuals’ evaluations of the technology (8).

Robotic surgery has diffused rapidly across healthcare systems worldwide, including China, despite limited public understanding of its benefits (9). This knowledge gap may not impede diffusion at the system level, yet it can strongly shape public perceptions and individual acceptance of the technology (10). Notably, existing research has often overlooked the public as a key stakeholder in medical technology adoption. As final decision-makers, individuals’ willingness to accept RS is as critical to its broader adoption as technical maturity itself (11, 12). The Unified Theory of Acceptance and Use of Technology (UTAUT) provides a systematic explanatory framework for analyzing this adoption process. Its original formulation does not fully explain decision-making in high-risk medical contexts (13–15). Prior studies have shown that trust and perceived risk are integral to predicting public acceptance of robotic surgery (16–18). These findings suggest that the conventional UTAUT model does not fully capture the psychological mechanisms involved in this setting. Behavioral intention is therefore shaped by more than rational evaluation alone.

A prevailing assumption in robotic surgery research is that public perceptions of surgical outcomes are largely consistent with clinical reality. While empirical evidence confirms that these perceptions usually diverge from actual clinical performance (19), most studies still examine subjective appraisals and objective outcomes separately. Few studies quantify the discrepancy between them or assess its influence on behavioral intention. In practice, information asymmetry between physicians and patients often reinforces this gap. While information asymmetry is common in specialized medicine, the manifestation of the perception-reality gap (PRG) may vary across cultural contexts. In China, hierarchical doctor-patient relationships and medical paternalism can amplify information barriers. These gaps are further widened as women navigate complex trade-offs between personal health, family responsibilities, and societal expectations (20). Such sociocultural pressures may further widen the mismatch between subjective perceptions and clinical realities, as behavioral decision theory suggests that decision-making is shaped by both cognitive capacity and environmental factors (21, 22). This implies that the perception-reality gap represents not merely statistical deviation but critical contextual factors shaping decision logic.

Accordingly, this study examines public willingness to adopt robotic-assisted gynecologic surgery from two perspectives. First, it investigates how subjective perceptions of postoperative outcomes influence behavioral intention. Second, it examines whether variation in the perception-reality gap are associated with different decision pathways across groups. The magnitude of the gap determines the extent to which psychological factors influence behavioral intention. By contrast, the direction of the gap reflects distinct cognitive patterns and decision logics. Treating respondents as a homogeneous group and applying a single structural model may obscure important group differences. Based on these considerations, the present study addresses two core research questions: RQ1: What are the primary determinants of Chinese women’s willingness to adopt RAGS? RQ2: Does the PRG among Chinese women alter the pathways through which these factors influence behavioral intention toward RAGS? If so, how?

This study contributes at both theoretical and practical levels. Theoretically, it extends the UTAUT framework by integrating trust, perceived risk, and individuals’ perceived postoperative difference (PPD). This extension enhances its explanatory power for technology acceptance in high-risk medical contexts. This study introduces a novel comparative approach that directly links subjective postoperative perceptions to clinical benchmark data, enabling a systematic quantification of the perception-reality gap. Using PRG as the basis for group classification, the study address a gap in the literature by clarifying how cognitive mismatches give rise to group-level differences in decision pathways. Practically, the findings offer actionable insights for medical institutions, policymakers, and technology developers. They provide concrete empirical support for optimization doctor-patient communication, promoting informed decision-making, and facilitating the rational implementation of advanced surgical technologies.

## 2 Theoretical background

### 2.1 Major theoretical models in healthcare technology acceptance

Healthcare technology acceptance has evolved through diverse multiple traditions rather than a single dominant framework (23). Unlike technology suffusion in non-clinical settings, healthcare acceptance is a multi-stakeholder negotiation involving patients, clinicians, and organizations (24). It is embedded in contexts where patient safety, professional accountability, regulatory requirements, and data privacy are central concerns (25). Consequently, healthcare technology acceptance is rarely a purely voluntary or utility-driven decision. This has driven a theoretical shift from models centered on isolated health beliefs to more integrative socio-technical frameworks designed to account for the clinical, organizational, and interpersonal complexity of healthcare technology acceptance (26).

The Health Belief Model (HBM) was among the earliest and most renowned social cognition frameworks for explaining health-related behavior change. It conceptualizes acceptance primarily through internal health appraisals, including perceptions of susceptibility, severity, benefits, and barriers (27). While HBM effectively captures preventive psychology, it often neglects technical attributes (28). Modern clinical systems involve layers of complexity that HBM cannot fully map. As a result, scholars frequently integrate HBM with other theoretical frameworks (e.g., TAM, TPB, and UTAUT) to strengthen its explanatory power in healthcare technology research (28–30).

As digital technologies became more deeply embedded in clinical practice, the Technology Acceptance Model (TAM) emerged as a dominant framework for explaining technology adoption. TAM emphasizes two core determinants: perceived usefulness and perceived ease of use (31). Its appeal lies in its parsimony and strong predictive utility. However, its parsimonious structure often faces explanatory limitations in complex healthcare environments. Reviews by Holden and Rahimi et al. indicate that TAM predicts a substantial share of health IT acceptance, but often requires contextual extension (32, 33). This is because its utilitarian focus does not fully account the sensitive conditions under which medical technologies are adopted.

Medical choices are seldom fully autonomous. The Theory of Planned Behavior (TPB) addresses this by incorporating attitudes, subjective norms, and perceived behavioral control, thereby broadening the explanatory scope of technology acceptance (34). In healthcare, this perspective is valuable. Despite its wide application across diverse domains, TPB also has theoretical limitations. The framework assumes a relatively coherent and reasoned decision process, which may oversimplify the complexity of human behavior in clinical settings (34, 35). Furthermore, scholars have noted ambiguity in its social and control constructs (36). TPB is therefore strong in explaining social-intentional processes but less sensitive to technology characteristics.

To overcome the limitations of isolated frameworks, the Unified Theory of Acceptance and Use of Technology (UTAUT) emerged as a comprehensive architecture. Venkatesh et al. (37) developed UTAUT by synthesizing eight earlier theories. The model identifies four core determinants of technology acceptance and use: performance expectancy, effort expectancy, social influence, and facilitating conditions. The first three primarily shape behavioral intention, whereas facilitating conditions more directly support actual use. The model also proposes that age, gender, experience, and voluntariness of use moderate these relationships. UTAUT has been widely applied in healthcare technology research, and systematic reviews identify it, alongside TAM, as one of the most prevalent acceptance frameworks in the field (23).

### 2.2 Technology acceptance in robot-assisted surgery: rationale for UTAUT

Research on robot-assisted surgery has expanded rapidly. However, most studies still focus on clinical outcomes, safety, cost, and comparisons with conventional surgical approaches (38). Existing research on acceptance intention remains limited, especially patients and the public. Early work was concentrated in professional settings and was mainly grounded in UTAUT. BenMessaoud et al. (14) were among the first to adapt UTAUT to robotic-assisted surgery, using it to identify the barriers and facilitators that shaped surgeons’ adoption decisions. Subsequent studies extended this line to more specific professional contexts. Krishnan et al. (39) applied a UTAUT-based framework to examine the adoption of transoral robotic surgery. As the field matured, the focus shifted toward potential patients and the general public (40). In this stage, patient-centered studies began to diverge into two directions: some adopted TAM-based extensions, while others expanded broader acceptance frameworks. Kao et al. (16) combined TAM with trust to explain patient willingness. They found that perceived usefulness, ease of understanding, and physician trust significantly influenced intention. At the same time, some studies sought to adapt acceptance models to the characteristics of high-risk surgical contexts. UTAUT has shown value in explaining patients’ attitudes toward surgical robots (41, 42). While these approaches have enriched the field, the complexity of surgical decision-making calls for a more systematic and context-sensitive framework.

UTAUT overcomes the structural limitations of earlier acceptance frameworks. It moves beyond the attributes of the technology itself and incorporates social and contextual determinants of acceptance. Its four core constructs of UTAUT accurately map the psychological trade-offs in RAS. Performance expectancy reflects anticipated clinical value. Effort expectancy captures the cognitive burden of understanding robotic surgery. Social influence reflects the role of physicians and family members in treatment decisions. Facilitating conditions represent the institutional and procedural support that makes the technology seem accessible and safe. Most importantly, UTAUT offers superior predictive precision. Empirical evidence confirms that while earlier models like TAM typically account for 40% of the variance in behavioral intention, UTAUT elevates this explanatory power to nearly 70% (43). This stronger explanatory scope makes it a more suitable starting point for complex medical decisions.

UTAUT also offers a flexible and adaptable framework. This flexibility supports its application across different clinical settings and patient populations. It also allows the model to capture the different psychological mechanisms involved in medical technology acceptance. This is especially important in robot-assisted surgery, where behavioral intention may be shaped by factors beyond the original constructs, particularly trust and perceived risk (42). UTAUT therefore provides a strong core structure while allowing for context-specific extension, making it a suitable foundation for the present study.

### 2.3 Incorporating perceived postoperative difference (PPD) into UTAUT

In this study, perceived postoperative difference (PPD) represents the subjective comparison between RAGS and conventional LS. Rather than reflecting general attitudes, PPD captures specific judgments regarding perioperative and postoperative outcomes. It translates women’s perceptions into concrete. These outcomes domains include surgical effectiveness, complications, length of stay, operative time, and costs (44, 45). These indicators align with the benchmarks most frequently used in clinical comparisons. This definition is consistent with patient-centered decision research (45, 46). Evidence from gynecologic oncology confirms that women consider more than survival-related outcomes. They also prioritize outcome expectations that affect daily life (47). Potential patients and members of the public therefore do not usually interpret robotic surgery through technical principles or device design. These aspects are outside the range of what they can directly assess or control.

PPD also has a clear theoretical foundation. First, diffusion of innovation theory regards perceived relative advantage as an important determinant of new technology adoption (48). Decisions are rarely based on objective performance alone. Instead, they depend on whether a new option is perceived as superior to existing alternatives (49). Second, the Health Belief Model suggests that individuals’ subjective judgments about the benefits of an action can significantly shape their behavioral choices (50). PPD builds on these two traditions and adapts them to the context of surgical decision-making.

The introduction of PPD is especially necessary in the context of RAGS, given persistent public misconceptions. Existing studies show that patients and the public often have limited knowledge of robotic surgery. In gynecologic settings, many women cannot clearly distinguish between LS and robotic procedures (51). Misunderstandings also persist regarding the surgeon’s role. Some individuals even believe that the robot operates autonomously rather than under direct human control (11). These views are often formed under conditions of information asymmetry. In this context, PPD provides a focused way to capture how women interpret the likely consequences of RAGS. It also helps explain how these perceptions, whether accurate or misaligned, shape behavioral intention.

## 3 Hypothesis development

### 3.1 Performance expectancy (PE)

Performance expectancy (PE) is the belief that using a technology will improve task performance. In the UTAUT framework, PE is consistently the strongest predictor of behavioral intention (BI) (37, 52). Evidence from healthcare technologies likewise shows a positive association between PE and intention to adopt new tools (53, 54). In surgical robotics, qualitative findings indicate that PE is a central driver of adoption decisions (14). For women, the cosmetic appearance of postoperative scars is also a major concern (55). Robot-assisted approaches are associated with better cosmetic results and higher satisfaction (56). In the context of robot-assisted gynecologic surgery (RAGS), PE thus captures expectations of superior efficacy, faster recovery, and improved scar appearance. Hence, we hypothesize:

*H1*: PE positively influences women's behavioral intention (BI) to choose RAGS.

### 3.2 Effort expectancy (EE)

Effort Expectancy refers to users’ perception of how easy a technology is to understand and use (37). In this study, EE represents the extent to which the public perceives it easy to understand information related to RAGS and to adapt to preoperative procedures. Existing research indicates that the public generally has a limited understanding of the differences between traditional laparoscopic surgery and robotic surgery. Moreover, many people are unfamiliar with the actual process of robotic operations. This lack of understanding may suppress acceptance intentions. Conversely, when a technology is perceived as easier to understand and use, the public’s willingness to adopt it increases (8, 51, 57). In complex technological settings, perceived ease also reinforces performance-related beliefs: when a technology is easy to understand and use, individuals are more inclined to believe it can achieve better performance or effectiveness (58, 59) Thus, this study hypothesizes:

*H2*: EE positively influences women's behavioral intention (BI) to choose RAGS.

*H2a*: EE significantly influences PE in the context of RAGS.

### 3.3 Facilitating conditions (FC)

Facilitating conditions refer to the extent to which users perceive that adequate organizational and technical infrastructures are available to support their use of a particular technology (37). A review by Williams et al. (60) reports a consistent positive association between FC and behavioral intention (BI) across multiple studies. In healthcare information environments, adequate technical support, infrastructure, and resource availability enhance the public’s positive evaluation of medical technologies and directly strengthen their willingness to adopt them (15, 61). Concurrently, studies in health technology and mobile health (mHealth) research confirm that sufficient resource conditions positively influence users’ performance expectations regarding technological benefits (62). Accordingly, this study proposes the following hypotheses:

*H3*: FC positively influences women's behavioral intention (BI) to choose RAGS.

*H3a*: FC significantly influences PE in the context of RAGS.

### 3.4 Social influence (SI)

Social Influence refers to users’ perception of the extent to which important others believe that they should adopt a new technology (37). In healthcare, SI is recognized as an important factor affecting patients’ adoption of new medical technologies (63). Cao et al. (64) indicate that SI not only directly predicts BI but also positively affects individuals’ PE toward technology.

Within the Chinese cultural context, medical choices often reflect family deliberation, a pattern frequently linked to Confucian relational norms (65, 66). At the same time, online media and social networking platforms have become major channels shaping perceptions of medical technologies. Arishi et al. (8) reported that 36.9% of respondents first learned about robot-assisted surgery (RAS) through social media, and new-media use is closely associated with public health behaviors in China (67). Authoritative endorsements enhance source credibility and perceived certainty, thereby strengthening both expectations and acceptance (68). Taken together, family opinions, authority effect, and media influence all constitute critical dimensions of medical decision-making. Based on this logic, this study localized the SI construct to reflect these contextual features and proposes the following hypotheses:

*H4*: SI positively influences women's behavioral intention (BI) to choose RAGS.

*H4a*: SI significantly influences PE in the context of RAGS.

### 3.5 Trust (T) and perceived risk (PR)

Trust and Perceived Risk are key variables explaining why people may deviate from rational expectations in highly sensitive medical contexts (69). A high level of trust in physicians enhances patients’ willingness to adopt new diagnostic or treatment methods (70, 71). However, when trust in the medical provider is excessive, a trust transfer effect can weaken the evaluation of privacy, safety, and failure risks, thereby increasing patients’ performance expectancy of the technology (72). Nevertheless, risk factors exist independently. PR remains a crucial determinant that inhibits the adoption of new medical technologies (73). Clinical risks have long attracted the joint attention of both physicians and patients, while patients’ willingness to adopt new technologies is often hindered by psychological resistance due to uncertainty about unfamiliar procedures (74). Specifically, concerns about potential negative clinical outcomes can significantly suppress acceptance of the technology (75). Thus, this study hypothesizes:

*H5*: Trust positively influences women's behavioral intention (BI) to choose RAGS.

*H5a*: Trust positively influences PE in the context of RAGS.

*H5b*: Trust is negatively associated with PR.

*H6*: PR negatively influences women’s behavioral intention (BI) to choose RAGS.

### 3.6 Perceived postoperative difference (PPD)

Perceived relative advantage serves as a fundamental driver of technology adoption. When the public perceives RAGS to be superior to prior surgical options, preferences, and choices are likely to shift toward RAGS. However, subjective perceptions of “relative advantage” do not necessarily reflect clinical reality. Misunderstandings about the role and capabilities of robotic surgery are common (51). Patients tend to overestimate expected benefits and underestimate potential risks, and such misaligned perceptions can ultimately shape their treatment choices (76). Accordingly, we hypothesize:

*H7*: PPD positively influences women's behavioral intention (BI) to choose RAGS.

*H7a*: PPD positively influences PE in the context of RAGS.

## 4 Methods

### 4.1 Study design and data collection

This study employed a cross-sectional survey design, combining a structured questionnaire with hospital-based clinical data. The target population comprised the general female public in China, rather than patients alone, to capture a more representative profile of social perceptions. Before the formal survey, a pilot test with 152 respondents was conducted to assess the comprehensibility, reliability, and validity of the questionnaire items. Based on participants’ feedback and preliminary statistical results, the instrument was iteratively revised. Several redundant items were removed to improve its measurement quality, including one item from the FC, one from EE, two from SI, and one from the Trust dimension. The final questionnaire consisted of demographic questions and 31 measurement items, all rated on a five-point Likert scale. The complete list of questionnaire items, along with their sources, is provided in Supplementary Appendix 1. The revised questionnaire demonstrated improved measurement properties and met the criteria for large-scale data collection. Data were collected anonymously through both online and offline channels over 1 month, from July to August 2025, yielding a total of 546 valid responses.

Concurrently, electronic medical record (EMR) data were retrieved from a tertiary teaching hospital in China for radical hysterectomy (RH) cases from January 2019 to December 2023, identifying 536 cases in total, including 307 robot-assisted radical hysterectomies (RRH) and 229 laparoscopic radical hysterectomies (LRH). RH is a critical gynecologic oncologic procedure primarily indicated for early-stage invasive cervical cancer. Compared with simple or total hysterectomy, RH involves greater anatomical complexity, distinct perioperative risks, and a different recovery trajectory, and is therefore often used as a benchmark for evaluating the clinical value of new surgical platforms in gynecologic oncology (77). Accordingly, we selected the RH cohort—with its large sample size and strong representativeness—as the index procedure for robotic-assisted gynecologic surgery in this study.

This study was approved by the Institutional Review Board of Central China Normal University and adhered to the Declaration of Helsinki and applicable regulations. For the survey component, all participants provided informed consent and responses were collected anonymously. For the clinical component, retrospective and fully de-identified electronic medical records were obtained, with all personal identifiers removed before analysis and no patient contact or intervention involved.

### 4.2 Clinical benchmark data

To ensure comparability between women’s subjective perceptions and objective clinical evidence, we established a clinical benchmarking process based on the EMR system. The laparoscopic radical hysterectomy (LRH) group (*n* = 229) was used as the reference cohort to evaluate the relative performance of the robot-assisted radical hysterectomy (RRH) group (*n* = 307) across key perioperative outcomes, including postoperative complication rate, postoperative hospital stays, operative time, intraoperative blood loss, surgical treatment cost, and total hospitalization cost. These variables represent the objective indicators commonly used in gynecologic oncologic practice (44) and correspond directly to the six PPD dimensions in the perception survey. To ensure comparability and minimize potential confounding variables, the analysis was strictly limited to these two procedures, which are highly comparable in clinical practice in terms of surgical indications and patient composition. To comprehensively describe the central tendency and dispersion of the data, the mean, median, and standard deviation of each continuous outcome were reported for both groups. Reporting both mean and median values allows for a more robust estimation of the central location, particularly for potentially skewed variables such as intraoperative blood loss. Detailed descriptive statistics are presented in Table 1.

**Table 1:** Descriptive clinical outcomes for radical hysterectomy by approach.

Surgery group		RRH (*n* = 307)	LRH (*n* = 229)
Comp (%)		17.90%	26.20%
Stay	Mean ± SD	8.05 ± 2.91	7.69 ± 1.57
	M	7	7
Op_Time	Mean ± SD	158.94 ± 46.55	150.07 ± 37.13
	M	150	143
Blood (ml)	Mean ± SD	196.89 ± 136.77	258.44 ± 265.80
	M	200	200
Surg_Cost	Mean ± SD	28825.89 ± 1663.56	29305.86 ± 789.89
	M	28896.5	29072.5
Hosp_Cost	Mean ± SD	67668.07 ± 10517.85	68590.43 ± 8351.22
	M	65249.34	66872.8

The core objective of this analysis was to transform clinical indicators with different measurement scales into a standardized objective score that is aligned with the five-point PPD scale used in the perception survey. To this end, intergroup differences were expressed as effect sizes. For continuous outcomes (postoperative hospital stay, operative time, intraoperative blood loss, surgical treatment cost, and total hospitalization cost), effect sizes were estimated using the Standardized Mean Difference (SMD). To ensure robustness of findings, Hedges’ g was adopted as the statistical estimator. Compared with Cohen’s d, Hedges’ g provides a small-sample correction and is therefore more appropriate when the two groups have unequal sample sizes. It offers an unbiased estimate of effect size and is widely regarded as the gold standard for cross-study comparison (78, 79). For interpreting effect magnitude, we applied Cohen’s conventional thresholds (±0.2, ±0.5) as a more rigorous and sensitive mapping criterion to the corresponding five-point scale levels, to capture finer differences and avoid broad-range misclassification (80). All transformed objective scores are reported with their 95% confidence intervals (95% CI) to indicate the precision of the estimates. For the dichotomous outcome measure of complication rates, we assessed differences using the risk ratio (RR) between groups. RR intuitively reflects the relative risk of complications in the robotic surgery group compared with the reference group and serves as a standard epidemiological indicator in clinical research. A clinical threshold band (RR ≤ 0.80) was further used to map RR values to the corresponding levels of the PPD scale (81). Effect sizes and mapping results are presented in Table 2.

**Table 2:** Standardized effects and PPD-aligned scores.

Outcome	Value	95% CI	Benchmark Score
Comp (%)	0.683	(Robot vs. Lap)	5 = Significantly less
Stay	0.151	(−0.021, 0.322)	3 = No difference
Op_Time	0.207	(0.035, 0.379)	2 = Slightly more
Blood (ml)	−0.304	(−0.476, −0.132)	4 = Slightly less
Surg_Cost	−0.352	(−0.525, −0.180)	4 = Slightly less
Hosp_Cost	−0.095	(−0.267, 0.076)	3 = No difference

### 4.3 Gap construction

Building on the clinical benchmarking, we operationalized the Perception-Reality Gap (PRG) to test women’s cognitive bias regarding differences between RAGS and LS. The item-level gap for each clinical outcome k was defined as:


$$\mathrm{Ga}p_{k}=\mathrm{PP}D_{k}-\text{BenchmarkScor}e_{k}$$

where k indexes the six clinical outcome dimensions; $\mathrm{PP}D_{k}$ is the respondent’s five-point PPD rating for outcome *k*; $\text{BenchmarkScor}e_{k}$ is the mapped clinical benchmark on the same five-point PPD scale. Positive values indicate overestimation of RAGS, negative values indicate underestimation; $|\mathrm{Ga}p_{k}|$ denotes the deviation magnitude.

Conceptually, the perception–reality gap (PRG) defined in this study is distinct from expectation–performance disconfirmation (EPD) in consumer behavior theory (82). EPD compares pre-use expectations with post-use perceived performance. It is typically used to explain post-consumption satisfaction (83). By contrast, PRG focuses on the alignment between subjective beliefs and objective reality. Its reference point is not an individual’s prior expectation, but external clinical benchmarks derived from comparative evidence. PRG is also not a post-experience construct (38). It is designed for potential patients and members of the public who may form judgments about robotic surgery without direct clinical experience (11, 38). For this reason, PRG is better suited than EPD to capture whether beliefs about surgical outcomes are aligned or misaligned with empirical evidence.

Because respondents may show opposite directions across outcomes, we constructed an overall indicator to capture the average tendency. The six item-level gaps showed high internal consistency (Cronbach’s 𝛼 = 0.89), supporting treatment as indicators of a single latent construct. We therefore computed the unweighted mean of the six gaps as the overall perception-bias score, which avoids arbitrary weighting across heterogeneous outcomes. To examine RQ2, we stratified the sample by the degree of the gap, using a tertile split of the overall perception-bias score to classify respondents into Underestimation, Alignment, and Overestimation groups. This stratification captures both the direction and the extent of the gap and effectively characterizes perception heterogeneity in the sample.

### 4.4 Data analysis

All data analyses were conducted in IBM SPSS Statistics v27.0 and AMOS v27.0. Descriptive statistics and confirmatory factor analysis (CFA) were used to assess the instrument’s reliability and validity. Structural equation modeling (SEM) was employed to test the hypothesized path relationships. Based on the degree of the gap, multi-group SEM compared path coefficients across the Underestimation, Alignment, and Overestimation groups. Analysis of variance (ANOVA) and chi-square (*χ*^2^) tests were additionally used to profile psychological characteristics within each group. All statistical tests were two-tailed, with significance set at *p* < 0.05.

## 5 Results

### 5.1 Demographic results

A total of 624 questionnaires were collected. After excluding responses with insufficient completion time, failed attention checks, or aberrant patterns, 546 cases remained (87.5% completion rate). Sample demographics are presented in Table 3. Respondents were predominantly 25–39 years old (64.65%), a reproductive and career-active life stage in which many women are employed, entering marriage, and considering childbirth; surgical decisions are therefore particularly salient in this group. Educational attainment was relatively high, with 66.12% holding a bachelor’s degree or above. Urban or town residents accounted for 68.49%. Public familiarity with RAGS was limited: 17.77% reported being “very familiar,” whereas 16.85% reported being “completely unfamiliar.” These figures indicate that knowledge of RAGS among the general female public in China remains at an early stage.

**Table 3:** Demographic characteristics of the sample.

Characteristic	Categories	*n*	%
Age (years)	18–24	117	21.43
	25–29	150	27.47
	30–39	203	37.18
	40–49	61	11.17
	≥50	15	2.75
Education level	High school or below	80	14.65
	Junior college/Associate degree	105	19.23
	Bachelor’s degree	211	38.65
	Master’s degree or above	150	27.47
Residence	Urban	217	39.74
	Town	157	28.75
	Rural	89	16.3
	Other	83	15.2
Marital status	Single	275	50.37
	Married	226	41.39
	Divorced	45	8.24
Employment status	Student	79	14.47
	Self-employed	62	11.36
	Public-sector employee	69	12.64
	Private-sector employee	119	21.79
	Professional/Technician & Researcher	67	12.27
	Healthcare worker	21	3.85
	Service/Sales or Manual worker	49	8.97
	Homemaker	7	1.28
	Retired	5	0.92
	Other	61	12.45
Familiarity with RAS	Very familiar	97	17.77
	Relatively familiar	121	22.16
	Generally familiar	133	24.36
	Slightly familiar	103	18.86
	Completely unfamiliar	92	16.85

### 5.2 Measurement model

The reliability and validity assessments of the scale are summarized in Tables 4, 5. In the confirmatory factor analysis (CFA), all standardized factor loadings were above the 0.70 threshold, supporting indicator reliability. Cronbach’s alpha for all constructs surpassed 0.70, satisfying reliability criteria and indicating strong internal consistency. Composite reliability (CR) values were all greater than 0.70, and the average variance extracted (AVE) for each construct was above the recommended threshold of 0.50, supporting convergent validity.

**Table 4:** Reliability and convergent validity tests summary.

Construct	Items	Factor loading	Cronbach’s α	CR	AVE
Performance expectancy	PE1	0.732	0.856	0.857	0.545
	PE2	0.754			
	PE3	0.712			
	PE4	0.702			
	PE5	0.786			
Effort expectancy	EE1	0.764	0.778	0.779	0.541
	EE2	0.709			
	EE3	0.732			
Social influence	SI1	0.771	0.815	0.815	0.525
	SI2	0.705			
	SI3	0.765			
	SI4	0.65			
Facilitating conditions	FC1	0.727	0.77	0.771	0.528
	FC2	0.711			
	FC3	0.742			
Trust	T1	0.829	0.768	0.769	0.529
	T2	0.679			
	T3	0.663			
Perceived risk	PR1	0.871	0.899	0.899	0.691
	PR2	0.859			
	PR3	0.774			
	PR4	0.818			
Perceived postoperative difference	PPD1	0.849	0.892	0.887	0.573
	PPD2	0.646			
	PPD3	0.852			
	PPD4	0.87			
	PPD5	0.572			
	PPD6	0.7			
Behavioral intention	BI1	0.856	0.887	0.888	0.725
	BI2	0.855			
	BI3	0.843			

**Table 5:** Heterotrait-Monotrait ratio (HTMT).

Construct	PE	EE	SI	FC	T	PR	PPD	BI
PE	0.738							
EE	0.462	0.736						
SI	0.110	0.345	0.725					
FC	0.294	0.369	0.269	0.727				
T	0.463	0.54	0.293	0.314	0.727			
PR	0.094	0.066	0.317	0.105	−0.176	0.831		
PPD	0.144	−0.063	−0.382	−0.397	0.132	−0.407	0.757	
BI	0.471	0.518	0.118	0.266	0.511	−0.113	0.224	0.851

Discriminant validity was confirmed via the Fornell–Larcker criterion: the square root of each construct’s AVE was larger than its inter-construct correlations, ensuring independence among constructs. In addition, all Heterotrait–Monotrait ratio (HTMT) values were below 0.90, further supporting discriminant validity (84). The measurement model demonstrated overall good fit: The chi-square to degrees of freedom ratio (*χ*^2^/df) was 2.404; the comparative fit index (CFI) was 0.932; the Tucker–Lewis index (TLI) was 0.923; the root mean square error of approximation (RMSEA) was 0.051; and the standardized root mean square residual (SRMR) was 0.043, meeting commonly accepted thresholds in the literature.

### 5.3 Structural model analysis

The structural model fit was evaluated using multiple indices. Results indicate an adequate overall fit to the observed data. The *χ*^2^/df ratio was 2.404, below the threshold of 3.0 and suggesting a satisfactory model fit. The CFI was 0.932 and the TLI was 0.923, both exceeding the acceptable level of 0.90. RMSEA was 0.051, within the acceptable range below 0.08, and the SRMR was 0.054, well below the 0.08 threshold. The overall model fit indices collectively demonstrated the validity and applicability of the proposed theoretical model. Path analysis results for each hypothesis (Table 6) revealed multiple key determinants influencing women’s behavioral intention (BI).

**Table 6:** Structural model evaluation.

Hyp.	Path	Estimate	S.E.	C.R.	*p*	Remarks
H1	PE → BI	0.176	0.083	3.362	0.000^***^	Supported
H2	EE → BI	0.302	0.095	4.863	0.000^***^	Supported
H2a	EE → PE	0.292	0.063	4.464	0.000^***^	Supported
H3	FC → BI	0.142	0.088	2.475	0.013^*^	Supported
H3a	FC → PE	0.209	0.060	3.356	0.000^***^	Supported
H4	SI → BI	0.008	0.070	0.161	0.872	Not Supported
H4a	SI → PE	−0.029	0.048	−0.522	0.602	Not Supported
H5	T → BI	0.180	0.104	2.900	0.004^**^	Supported
H5a	T → PE	0.214	0.071	3.203	0.001^***^	Supported
H5b	T → PR	−0.148	0.082	−2.937	0.003^**^	Supported
H6	PR → BI	−0.046	0.040	−1.168	0.243	Not Supported
H7	PPD → BI	0.234	0.062	4.208	0.000^***^	Supported
H7a	PPD → PE	0.203	0.042	3.400	0.000^***^	Supported

Standardized path coefficients are reported. *p < 0.05, **p < 0.01, ***p < 0.001.

Among all predictor variables, Effort Expectancy (EE) demonstrated the strongest positive predictive effect on Behavioral Intention (BI) (*β* = 0.302, *p* < 0.001), supporting H2. This indicates that when evaluating complex and information-opaque medical technologies like robotic surgery, women’s behavioral decisions are significantly driven by their perceived cognitive costs. Simultaneously, EE also positively shaped their PE of the technology, supporting H2a (*β* = 0.292, *p* < 0.001). This suggests that when information is easier to comprehend and process, respondents are more likely to use it as a key reference for judging the advantages of a surgical technology.

Similarly, Performance Expectancy (PE) had a significant positive impact on BI, supporting H1 (*β* = 0.176, *p* < 0.001). This illustrates that respondents who believed RAGS offered higher clinical benefits demonstrate stronger adoption intentions, confirming the outcome-oriented decision logic commonly observed in medical decision-making.

Facilitation Conditions (FC) exerted significant positive effects on both BI and PE, supporting H3 (*β* = 0.142, *p* = 0.013) and H3a (*β* = 0.209, *p* < 0.001). This implies that adequate external resources support is not only a prerequisite for making choices, but their accessibility also enhances respondents’ positive expectations of RAGS.

However, the direct effects of Social Influence (SI) on both BI and PE were not significant, with H4 (*β* = 0.008, *p* = 0.872) and H4a (*β* = −0.029, *p* = 0.602) unsupported. This finding suggests that opinions from social networks or family did not directly influence participants’ decisions, indicating that individual beliefs about the surgery’s efficacy are less reliant on external social consensus.

Trust (T) played a pivotal role in the model, with all hypothesized pathways supported: H5 (*β* = 0.180, *p* = 0.004), H5a (*β* = 0.214, *p* = 0.001), and H5b (*β* = −0.148, *p* = 0.003). These results demonstrate that trust not only strengthens respondents’ confidence in the outcomes and adoption of RAGS but also alleviates their concerns about potential risks and uncertainties.

In contrast, the direct inhibitory effect of Perceived Risk (PR) on BI was not significant, and H6 was not supported (*β* = −0.046, *p* = 0.243). However, trust exerted a significant negative influence on PR, indicating that higher levels of trust are associated with lower perceived risk among respondents.

Regarding the self-constructed Perceived Postoperative Difference (PPD), it was confirmed to significantly and positively influence both BI and PE, supporting H7 (*β* = 0.234, *p* < 0.001) and H7a (*β* = 0.203, *p* < 0.001). This finding provides direct empirical evidence for the core argument of this study: subjective comparative outcomes form a crucial psychological basis for evaluating the value of robotic-assisted surgery. Women who perceive the expected outcomes of robotic gynecological surgery as more advantageous are more likely to choose this option.

**Figure 1:**
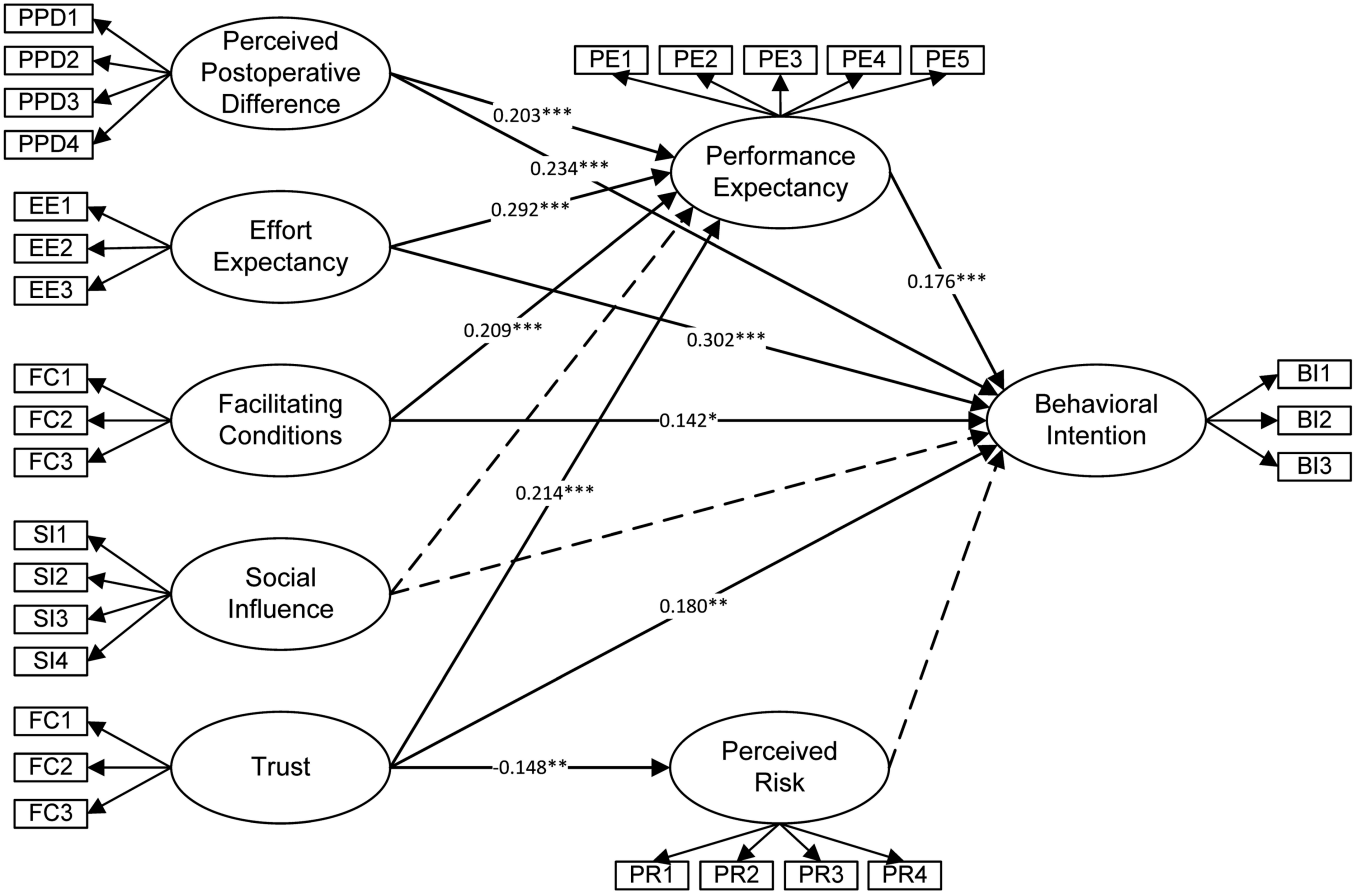
Structural equation model results. Solid lines represent significant paths (*p* < 0.05); dashed lines represent non-significant paths.

Overall, these results reveal that in a medical context characterized by both information asymmetry and a culture of trust orientation, Chinese women’s adoption intentions toward RAGS is jointly driven by PE, EE, FC, T, and PPD. These findings offer valuable insights for improving patient-provider communication, optimizing medical resource allocation, and promoting the appropriate dissemination of advanced surgical technologies. The final structural equation model is presented in Figure 1.

### 5.4 Multi-group analysis

To examine differences in adoption mechanisms across varying levels of perception–reality gap, respondents were divided into three groups: the underestimation group (*n* = 179), the alignment group (*n* = 184), and the overestimation group (*n* = 183). These groups represent the degree of consistency between respondents’ subjective perceptions and the objective clinical outcomes. Each group’s sample size exceeded the minimum requirement for Structural Equation Modeling (SEM). Measurement invariance tests demonstrated satisfactory results. Metric invariance was supported (ΔCFI ≤ 0.01), indicating that the three groups were comparable in terms of factor loadings. Structural path invariance exceeded the threshold, confirming significant path differences between groups and thus meeting the conditions for multi-group comparison analysis.

Results of between-group path comparisons are presented in Table 7. Group differences were assessed based on the Critical Ratio for Differences (C.R.), which follows a standard normal distribution. Path differences were considered significant when the absolute value of C.R. exceeded 1.96, corresponding to a significance level of *p* < 0.05 (85).

**Table 7:** Between-group path differences.

Path	β (G1)	β (G2)	β (G3)	G1 vs. G2	G1 vs. G3	G2 vs. G3
PE → BI	0.252**	0.057	−0.069	−1.35	−2.32*	−0.83
EE → BI	0.199	0.072	0.487***	−1.00	2.12*	2.87**
EE → PE	0.335*	−0.026	0.222	−1.22	2.45*	2.10*
FC → BI	0.208	0.191	0.088	−0.25	−0.59	−0.47
FC → PE	0.010	0.263*	0.106	−0.53	−0.84	−0.51
SI → BI	0.276*	−0.031	−0.277**	−2.23*	−3.43***	−2.17*
SI → PE	−0.146	0.045	−0.176	−2.05*	−2.88**	−1.44
T → BI	0.243	0.151	0.035	−0.67	−1.05	−0.61
T → PR	0.407***	−0.181*	−0.378***	−0.82	−2.64**	−1.73
T → PE	0.420*	0.247*	−0.093	−0.94	−1.58	−0.76
PR → BI	0.001	−0.052	−0.196*	−0.47	−1.72	−1.30
PPD → BI	0.102	0.344	−0.066	1.47	−0.74	−1.67
PPD → PE	−0.197	0.343	0.399***	1.88	2.44*	1.21

|C.R.| > 1.96, *p < 0.05; |C.R.| > 2.58, **p < 0.01; |C.R.| > 3.29, ***p < 0.001.

Values with |C.R.| < 1.96 are considered non-significant.

G1 = Underestimation group; G2 = Alignment group; G3 = Overestimation group.

The comparison results showed that the effects of EE on both BI and PE were significantly stronger in the overestimation group than in the other groups (|C.R.| ≈ 2.10–2.87, *p* < 0.05–0.01). This suggests that when respondents hold an optimistic view of robotic-assisted gynecologic surgery, easily understood and adaptable surgical processes are perceived as indicators of better outcomes, which in turn strengthens their intention to adopt the technology.

Conversely, the effects of SI on both BI and PE were significantly weaker in the overestimation group (|C.R.| ≈ 2.05–3.43, *p* < 0.05–0.001), and even negative in some cases. This indicates that when individuals are overly confident in the technology’s performance, social influence no longer serves as a facilitating factor and may even diminish adoption willingness due to external skepticism.

The mitigating effect of T on PR was also significantly stronger in the overestimation group (|C.R.| = 2.64, *p* < 0.05–0.01). This implies that under optimistic perceptions, higher trust effectively reduces perceived risk and enhances individuals’ confidence in adopting robotic-assisted gynecologic surgery.

Finally, the positive effect of PPD on PE was primarily observed in the alignment and overestimation groups, with the strongest effect found in the latter. The path difference between the underestimation and overestimation groups was significant (|C.R.| = 2.44, *p* < 0.05), while the difference between the underestimation and alignment groups was marginally significant (|C.R.| = 1.88).

In the underestimation group, BI was mainly influenced by two positive predictors: PE (*β* = 0.252, *p* = 0.005) and SI (*β* = 0.276, *p* = 0.022). This indicates that individuals who underestimate the benefits of robotic-assisted gynecologic surgery rely more on perceived clinical benefits and others’ opinions when forming their willingness to adopt the procedure.

In the alignment group, although none of the direct paths from predictors to Behavioral Intention (BI) reached statistical significance, several other significant relationships were observed. FC had a significant positive effect on PE (*β* = 0.263, *p* = 0.013), while Trust (T) significantly enhanced PE (*β* = 0.247, *p* = 0.024) and significantly reduced PR (*β* = −0.181, *p* = 0.039). These results indicate that respondents whose perceptions were closely aligned with clinical reality had already formed relatively stable and rational cognitive structures, meaning they were less likely to adjust their behavioral intentions based on single changes in perception.

In the overestimation group, EE had the strongest positive influence on BI (*β* = 0.487, *p* < 0.001), suggesting that individuals with higher perceived advantages of robotic-assisted gynecologic surgery placed greater emphasis on the convenience and accessibility of the surgical process. Conversely, SI significantly suppressed BI (*β* = −0.277, *p* = 0.006), indicating that greater external pressure or opposing opinions tended to trigger negative reactions, thereby reducing willingness to choose robotic-assisted gynecologic surgery. Similarly, PR also exerted a significant negative effect on BI (*β* = −0.196, *p* = 0.019), suggesting that decision-making in this group was more directly affected by concerns about potential adverse outcomes—an effect not observed in the other two groups.

Regarding other intermediate paths, the effect of PPD on PE was significantly positive only in the overestimation group (*β* = 0.399, *p* = 0.001), suggesting that for these respondents, expectations of surgical outcomes were shaped primarily by subjective perceptions rather than objective evidence. Trust (T) also had a significant influence on PR in both the underestimation group (*β* = 0.407, *p* = 0.044) and the overestimation group (*β* = −0.378, *p* < 0.001), although in opposite directions. This finding indicates that when surgical outcomes are overly overestimated, trust more strongly reduces perceived risk.

### 5.5 Group-level differences

Based on the three groups categorized by their perception-reality gap, this study employed one-way Analysis of Variance (ANOVA) to compare the mean scores of the core constructs across the groups (Table 8).

**Table 8:** Group differences in core construct scores.

Construct	F	*p* value	η^2^	*Post Hoc* tests
BI	36.33	<0.001	0.118	G3 > G1 ≈ G2
PE	17.47	<0.001	0.060	G3 > G1 ≈ G2
EE	6.13	0.002	0.022	G2 < G1 ≈ G3
FC	15.17	<0.001	0.053	G1 > G2 ≈ G3
SI	22.64	<0.001	0.077	G1 > G2 > G3
PR	36.39	<0.001	0.118	G1 > G2 > G3
T	9.09	<0.001	0.032	G3 > G2 > G1

η^2^ values represent effect sizes, interpreted following Cohen (1988): small ≈ 0.01, medium ≈ 0.06, large ≈ 0.14 (80).

*Post hoc* tests were determined using Tukey or Games–Howell tests according to Levene’s test results. All F-tests are significant at *p* < 0.01.

G1 = Underestimation group; G2 = Alignment group; G3 = Overestimation group.

Before the ANOVA, data normality was assessed using the Shapiro–Wilk test, and homogeneity of variances was verified using Levene’s test. The Games–Howell *post-hoc* test was applied for variables that violated the assumption of variance homogeneity, while Tukey’s HSD test was used for the remaining variables.

The results revealed significant inter-group differences for all constructs (*p* < 0.01), with effect sizes (η^2^) ranging from 0.022 to 0.118. This confirms that the perception-reality gap has substantial explanatory power for individuals’ behavioral assessments. The inter-group differences for BI and PR were the most pronounced (η^2^ = 0.118), approaching Cohen’s threshold for a large effect (η^2^ ≈ 0.14). This indicates that the perception-reality gap strongly differentiates participants’ adoption intentions and risk perceptions. Conversely, the effect size for EE was the smallest (η^2^ = 0.022), indicating a smaller, though still significant, magnitude of difference.

The Overestimation group (G3) scored significantly higher on BI and PE, indicating that an overly optimistic cognitive tendency is closely associated with stronger confidence and adoption intention. This group also reported significantly higher T than the other two groups, reflecting a high level of confidence in both the robotic technology and the medical system.

In contrast, the Underestimation group (G1) scored highest on FC, SI, and PR, suggesting this cohort is more reliant on external social support and is characterized by greater risk sensitivity. The Aligned group (G2) generally fell in the middle, consistent with its positioning as the rational baseline. Notably, this group scored lowest on EE, perhaps reflecting a more pragmatic and realistic assessment of the costs associated with using robotic gynecological surgery.

Overall, these results demonstrate that the perception-reality gap systematically shapes individuals’ cognitive, affective, and behavioral evaluation patterns, distinguishing three distinct psychological profiles: optimistic (G3), rational (G2), and cautious (G1).

Further chi-square (*χ*^2^) tests (Table 9) revealed significant associations between respondents’ demographic characteristics and the perception–reality gap groups. Age group (*χ*^2^ = 18.29, *p* = 0.019, V = 0.129), educational level (*χ*^2^ = 24.81, *p* = 0.002, V = 0.151), marital status (*χ*^2^ = 9.56, *p* = 0.048, V = 0.094), and employment status (*χ*^2^ = 107.09, *p* < 0.001, V = 0.313) were all significantly related to group membership. Respondents with higher education levels and technical or professional occupations were more likely to fall into the overestimation group, whereas those with lower educational attainment or unstable employment were more frequently distributed in the underestimation group. This indicates that the formation of cognitive biases among respondents is influenced not only by psychological factors but also by underlying sociodemographic conditions.

**Table 9:** Demographic characteristics across groups.

Demographic variable	*χ* ^2^	df	*p* value	Cramer’s V
Age (years)	18.29	8	0.019	0.129
Education level	24.81	8	0.002	0.151
Marital status	9.56	4	0.048	0.094
Employment status	107.09	18	0.000	0.313

## 6 Discussion

This study extends the UTAUT framework and, innovatively, links women’s subjective perceptions with objective gynecologic clinical benchmarks. We show that subjective evaluations of postoperative outcomes are a key psychological process shaping behavioral intention (BI), and we confirm that the perception–reality gap significantly shapes the pathways through which women form surgical decisions. By delineating how members of the Chinese female public form intentions to choose robotic-assisted gynecologic surgery (RAGS), the study offers a new perspective on decision logic in the context of emerging medical technologies. The findings extend theory on healthcare technology adoption and provide practical guidance for designing more targeted communication between doctors and patients and for presenting information more effectively, thereby supporting the appropriate adoption and use of advanced robotic gynecologic surgery.

### 6.1 Determinants of behavioral intention

First, Chinese women’s acceptance of Behavioral Intention (BI) is strongly influenced by Effort Expectancy (EE). When the perceived cognitive cost of understanding RAGS is lower, women’s self-efficacy increases, which raises the likelihood of forming a positive intention. Greater comprehensibility eases the anxiety that accompanies high-risk medical choices and makes acceptance more likely. Furthermore, EE enhances Performance Expectancy (PE) by strengthening favorable evaluations of surgical performance, consistent with prior research showing that ease of understanding is a precondition for positive performance beliefs (59). In Chinese clinical practice, although tiered care has been implemented, most women still seek large hospitals for complex gynecologic procedures, where consultation time is limited and patients must finalize plans and complete preoperative preparation within short visits. When women receive clear and structured surgical information, they can form a more complete understanding during consultations, which in turn elevates performance expectations and strengthens their intention to choose RAGS. This finding is supported by (86, 87), which reported that multimedia supplements for preoperative education significantly enhance acceptance among patients lacking background knowledge of robotic surgery. Our findings further extend this perspective by positioning effort expectancy (EE) as the fundamental psychological mechanism.

Perceived Postoperative Difference (PPD) is an important determinant of both BI and PE. Among the significant predictors, the effect of PPD on BI was second only to EE, indicating that the public tends to act on subjective criteria that they construct about outcomes. Faced with specialist knowledge barriers, individuals often compare procedures using their perceived postoperative indicators. When women believe that robotic-assisted gynecologic surgery is superior to conventional approaches, this perceived advantage not only directly increases their willingness to adopt the technology but also provides a cognitive basis for higher performance expectations. This pattern is consistent with prior theory, which identifies perceived relative advantage as a strong predictor of behavior change (48, 50).

The positive effect of PE on BI is in line with the UTAUT framework (37), implying that beliefs about technical effectiveness are a key driver of adoption among Chinese women. More importantly, beyond EE and PPD, PE is also shaped by Facilitating Conditions (FC) and Trust (T). When FC is adequate, the probability that the technology’s potential benefits will be realized increases. Accordingly, respondents reasonably infer that having the necessary preconditions in place before implementation, such as adequate infrastructure and a well-trained, experienced team, will improve the quality of robotic-assisted gynecologic surgery. The effect of T on PE further suggests that when respondents believe medical professionals recommend the technology based on scientific judgment and the public interest, they are more likely to view the technology as genuinely valuable, and to attribute favorable outcomes to a stable platform and capable clinical teams.

Facilitating Conditions (FC) also significantly influence BI. In China, advanced medical technologies have expanded rapidly but remain unevenly distributed. Before making a treatment decision, patients commonly assess local resource availability, which is a fundamental feasibility check. Although robotic-assisted surgery has grown quickly, availability still varies by region. Institutions with robotic platforms and systematically trained teams are concentrated in a limited number of large tertiary hospitals. As a result, assessing FC naturally becomes a first step in decision-making. The public weighs whether local hospitals have the requisite infrastructure, technical capacity, and information support to judge whether adopting such advanced technology is truly feasible.

This study also finds a dual effect of Trust (T) on BI and Perceived Risk (PR). Trust is essential for lowering perceived risk and uncertainty associated with technology adoption, and it directly increases women’s willingness to accept robotic-assisted gynecologic surgery. These results are consistent with prior work (70, 71, 88). On the one hand, members of the public often associate hospitals that can perform robotic surgery with stronger clinical competence; this quality signal amplifies the positive influence of trust on choice. On the other hand, because patients cannot directly monitor the operative process and cannot rely on their own knowledge to evaluate potential hazards, trust becomes the key mechanism for reducing perceived risk. This aligns with evidence that trust effectively attenuates risk perceptions in settings of technological uncertainty (72).

However, the paths from Social Influence (SI) to BI and to PE, as well as the path from PR to BI, did not reach statistical significance. This suggests that when knowledge of robotic-assisted gynecologic surgery is limited, social influence and potential risks alone are insufficient to determine choice. Although some respondents do attend to recommendations from important others, as the group analyses later corroborate, such influence does not necessarily alter their willingness to choose the procedure. The non-significant effect of PR on BI may indicate that, in considering robotic-assisted gynecologic surgery, the public focuses more on anticipated benefits than on risks. Many respondents view hospitals offering these procedures as institutions with high safety standards, which weakens the subjective impact of risk. This finding underscores the need to strengthen risk communication, particularly by providing transparent adverse-event data and postoperative recovery information, so that the public can form a more balanced risk perception and make informed, rational decisions. Notably, a separate investigation found a contrasting pattern: fear of errors showed the highest negative correlation with willingness, higher perceived risk-related concerns were associated with lower willingness to participate (89). This divergence may reflect sample differences or suggest that risk perceptions become salient when measured through concrete concerns rather than general risk constructs.

### 6.2 Group differences based on the perception-reality gap

Multi-group analysis shows that different levels of the perception–reality gap shift the information that women prioritize when making choices, leading to significant between-group differences in the same structural paths. In the overestimation group, Effort Expectancy (EE) is the primary driver of Behavioral Intention (BI), indicating greater emphasis on the convenience and ease of the surgical process. By contrast, Social Influence (SI) significantly suppresses BI in this group. This counterintuitive result suggests a central role for psychological reactance. In the standard UTAUT model, SI typically guides and positively affects BI. However, for women who hold overly optimistic expectations about the technology, external encouragement to choose a particular option can be perceived as intrusive, triggering reactance and reducing their willingness to adopt the procedure.

Group composition helps to explain this pattern. As shown in the demographic analyses, the overestimation group contains a higher proportion of women with higher educational attainment and technical or professional occupations. It also includes a relatively larger share of younger respondents, particularly those aged 18–24 years. With broader information access and stronger cognitive confidence, these respondents are more inclined to make autonomous choices in major medical decisions. For younger women, robotic surgery may still feel somewhat distant from their immediate medical decision-making. This can make their evaluations more optimistic and less grounded in concrete clinical trade-offs. Prior studies have shown that women’s autonomy in healthcare decision-making is associated with both occupation and education (90, 91). Thus, when strong recommendations are voiced by members of the social network—especially male family members or older relatives—such guidance may be interpreted as a challenge to one’s judgment and independence, provoking resistance and lowering BI (92).

Moreover, the negative effect of Trust (T) on Perceived Risk (PR) is strongest in the overestimation group. Under optimistic perceptions, trust functions as a core condition for maintaining low perceived risk, implying that within this group, trust is treated as a necessary precondition for how surgical risk is construed.

In sharp contrast, the underestimation group showed BI driven primarily by Performance Expectancy (PE) and Social Influence (SI) in the positive direction. This pattern indicates that when women hold a conservative view of robotic-assisted gynecologic surgery, their choices rely more on verifiable expectations of efficacy and on endorsement from the social environment. Mean-difference results further suggest that, within this group, higher PR creates a highly uncertain decision context. Under such information-poor, high-threat conditions, SI functions as a substitute information source. Demographically, this group is strongly associated with lower educational attainment and unstable employment, underscoring structural disparities in the diffusion of health information. This pattern was also identified by prior studies (51), who reported that women with lower educational attainment had significantly poorer understanding of surgical approaches, but our findings build on this by showing how such knowledge gaps translate into distinct decision pathways. In China, medical information resources are unevenly distributed, and health literacy varies markedly. For resource-constrained respondents, difficulties in accessing and interpreting complex clinical data foster uncertainty; consequently, women in the underestimation group tend to seek external support to bridge knowledge gaps, treating positive messages from authorities, relatives, or media as social proof of procedural reliability and using such signals as a basis for choice.

Women in the alignment group display the most stable and balanced decision structure. Their subjective perceptions closely match objective information, indicating a fuller understanding, stronger cognitive capacity, and a broader informational base. This group also included a relatively larger proportion of respondents aged 25–29 years. A possible explanation is that women in this age range often have more diverse channels for obtaining information, particularly through mobile internet use and social media. Greater exposure to health- and technology-related information may help them form more balanced judgments about robotic surgery. Although no single predictor showed a direct effect on BI in this group, Facilitating Conditions (FC) and T both significantly increased PE. Trust also reduced PR in this group, suggesting that when individuals have adequate information and can appraise it objectively, they are more likely to reach informational consensus with providers and to view the health system as capable of controlling and reducing surgical risk. The alignment group thus reflects a rational, multi-criteria decision mode that approximates an ideal state of new-technology acceptance.

Whether this pattern is culture-specific should be considered with caution. Group differences may be more pronounced in the Chinese context, where medical decision-making is shaped by doctor–patient information asymmetry, the prevailing structure of clinical authority, and the involvement of family members. However, this pattern should not be reduced to a merely local phenomenon. The broader mechanism identified in this study is unlikely to be uniquely Chinese. More broadly, the findings suggest that the same core acceptance mechanism can take different forms depending on how individuals process information and situate themselves within a given social and clinical context. In this sense, cross-cultural differences are likely to arise not from different underlying mechanisms, but from differences in the relative weight and expression of the same component pathways.

### 6.3 Theoretical implications

In conclusion, this study validated the key determinants of robotic gynecological surgery adoption among Chinese women, demonstrating that Postoperative Perception Difference (PPD) is a critical and independent determinant of behavioral intention (BI). Our principal finding, however, is that the Perception-Reality Gap (PRG) is not merely a cognitive error but a pivotal background context that dictates essential differences in decision-making logic. The multigroup analysis revealed how the PRG reshapes the formation paths of behavioral intention. Furthermore, the PRG showed a significant correlation with demographic characteristics. This finding reveals that individuals with higher education or technical professions tend to over-estimate surgical advantages, while those with lower education or less stable occupations tend to under-estimate its value, reflecting underlying socio-structural disparities.

The theoretical contribution of this study lies in moving beyond descriptive research and studies that merely identify factors influencing acceptance of robotic surgery, revealing the underlying decision-making mechanisms shaped by the perception–reality gap (PRG). Specifically, we demonstrate that the determinants of behavioral intention operate through different pathways across the subgroups defined by the perception-reality gap. This finding advances the research perspective from examining what factors predict acceptance to investigating how these factors function differently across populations with distinct cognitive starting points, thereby providing a theoretical foundation for precision communication strategies in clinical practice.

These findings, linked to the realities of social resource distribution in China, have broad practical significance and provide an empirical foundation for developing precise public communication strategies. Specifically, for the overestimation group, clinical communication must focus on expectation management and risk calibration to foster rational, evidence-based cognition. For the aligned group, healthcare providers should ensure full transparency, offering comprehensive and balanced information to respect and facilitate autonomous choice. Lastly, for the underestimation group, the priority is to bolster confidence and reduce uncertainty by providing authoritative evidence and building social support, such as presenting objective data on the advantages of robotic surgery during consultations.

## 7 Limitations and future considerations

This study has several limitations, which in turn provide directions for future research.

First, this study was conducted within a specific cultural and healthcare context, which may limit the cross-cultural generalizability of the findings. Although the core acceptance mechanism identified here may operate across settings, its component pathways may differ in relative weight and configuration under different cultural and institutional conditions. Future research could replicate this study in other countries and healthcare systems to test the stability of the model across contexts. Second, the measurement of the social influence construct was limited. Specifically, items intended to capture concepts such as peer opinions and conformity effects failed to meet the required thresholds for reliability and validity. Future research could employ qualitative interviews or a mixed-methods approach to further investigate these relationships. Finally, this study relied on self-reported cross-sectional survey data, which may introduce biases. Incorporating behavioral or longitudinal measurements in future work could strengthen the robustness of the conclusions.

## Data Availability

The datasets used in this study contain anonymized survey responses and de-identified hospital records that cannot be publicly shared due to institutional and privacy restrictions. The data are available from the corresponding author upon reasonable request and with approval from the Institutional Review Board. Requests to access the datasets should be directed to Zhengzijin Yang, yangzzj@mails.ccnu.edu.cn.
