# Supplementary material for: Bridging perception and reality: understanding Chinese women’s behavioral intentions toward robot-assisted gynecologic surgery
Source: Front Public Health. 2026 Apr 2;14:1743010. doi: 10.3389/fpubh.2026.1743010 (PMC13083124; doi:10.3389/fpubh.2026.1743010)
Supplement: Supplementary file 1 [file Table_1.docx]

**Supplementary Appendix 1. Survey Instrument**

| Construct | Item | Source |
| --- | --- | --- |
| Performance expectancy  （PE） | PE1: I believe robot-assisted gynecologic surgery can improve surgical treatment outcomes. | [1-4] |
|  | PE2: I believe robot-assisted gynecologic surgery enables faster postoperative recovery. |  |
|  | PE3: I believe robot-assisted gynecologic surgery will reduce the occurrence of surgical complications. |  |
|  | PE4: I believe robot-assisted gynecologic surgery leads to smaller and more cosmetically appealing scars. |  |
|  | PE5: I believe the clinical efficacy of robot-assisted gynecologic surgery is superior to that of conventional laparoscopic. |  |
| Effort expectancy  （EE） | EE1: It is easy for me to understand information about robot-assisted gynecologic surgery. | [1] |
|  | EE2: I can adapt to the procedures and preparations required for robot-assisted gynecologic surgery. |  |
|  | EE3: I can easily understand how robot-assisted gynecologic surgery differs from other surgical approaches. |  |
| Social influence  （SI） | SI1: Recommendations from physicians influence my choice of surgical approach. | [1, 8] |
|  | SI2: Family members’ opinions influence my choice of surgical approach. |  |
|  | SI3: Endorsement from industry authorities or professional organizations influences my choice of surgical approach. |  |
|  | SI4: Media coverage and public opinion about robotic surgery influence my choice. |  |
| Facilitating conditions  （FC） | FC1: I can obtain information on robot-assisted gynecologic surgery from multiple sources easily. | [1] |
|  | FC2: Hospitals I am familiar with provide the resources needed to offer robot-assisted gynecologic surgery. |  |
|  | FC3: I believe local hospitals have the technical capability to perform robot-assisted gynecologic surgery. |  |
| Trust  （T） | T1: I trust that hospitals can perform robot-assisted gynecologic surgery safely. | [1, 3] |
|  | T2: I trust physicians to recommend a surgical approach that suits my needs. |  |
|  | T3: I trust physicians to perform robot-assisted gynecologic surgery safely and competently. |  |
| Perceived Risk  （PR） | PR1: I am concerned that robot-assisted gynecologic surgery may have technical malfunctions. | [1, 2] |
|  | PR2: I worry that emergency response during robot-assisted gynecologic surgery may be limited in unexpected situations. |  |
|  | PR3: Compared with conventional laparoscopy, I perceive robot-assisted gynecologic surgery as riskier. |  |
|  | PR4: I am concerned about adverse outcomes that may result from robot-assisted gynecologic surgery. |  |
| Perceived Postoperative Difference（PPD） | PPD1 (StayΔ): Compared with conventional laparoscopy, how is the length of hospital stay with robot-assisted gynecologic surgery? | [1, 5, 6, 7] |
|  | PPD2 (OTΔ): Compared with conventional laparoscopy, how is the operative time with robot-assisted gynecologic surgery? |  |
|  | PPD3 (BloodΔ): Compared with conventional laparoscopy, how is intraoperative blood loss with robot-assisted gynecologic surgery? |  |
|  | PPD4 (CompΔ): Compared with conventional laparoscopy, how is the complication rate with robot-assisted gynecologic surgery? |  |
|  | PPD5 (SurgCostΔ): Compared with conventional laparoscopy, how are procedure-related costs with robot-assisted gynecologic surgery? |  |
|  | PPD6 (HospCostΔ): Compared with conventional laparoscopy, how are hospitalization costs with robot-assisted gynecologic surgery? |  |
| Behavioral intention（BI） | BI1: I am willing to undergo RAS. | [1-3] |
|  | BI2: If clinically appropriate and feasible, I would choose RAS. |  |
|  | BI3: I would recommend RAS to others. |  |

**Reference**

[1] Venkatesh, V., Morris, M. G., Davis, G. B., & Davis, F. D. (2003). User acceptance of information technology: Toward a unified view. MIS quarterly, 425-478.

[2] de Andres-Sanchez, J., Almahameed, A. A., Arias-Oliva, M., & Pelegrin-Borondo, J. (2022). Correlational and configurational analysis of factors influencing potential patients’ attitudes toward surgical robots: A study in the Jordan university community. Mathematics, 10(22), 4319.
[3] Kao, H.-Y., Yang, Y.-C., Hung, Y.-H., & Wu, Y. J. (2022). When does Da Vanci robotic surgical systems come into play? Frontiers in Public Health, 10, 828542.

[4] Krishnan, G., Mintz, J., Foreman, A., Hodge, J. C., & Krishnan, S. (2019). The acceptance and adoption of transoral robotic surgery in Australia and New Zealand. Journal of Robotic Surgery, 13(2), 301-307.

[5] BenMessaoud, C., Kharrazi, H., & MacDorman, K. F. (2011). Facilitators and barriers to adopting robotic-assisted surgery: contextualizing the unified theory of acceptance and use of technology. PloS one, 6(1), e16395.

[6] Wright, J. D., Ananth, C. V., Lewin, S. N., Burke, W. M., Lu, Y. S., Neugut, A. I., ... & Hershman, D. L. (2013). Robotically assisted vs laparoscopic hysterectomy among women with benign gynecologic disease. Jama, 309(7), 689-698.

[7] Shukla-Kulkarni, A., & Sethi, N. (2024). What Patients Think About Robot-Assisted Surgery: Lessons to Learn from the Awareness and Perception Study in Mumbai. The Journal of Obstetrics and Gynecology of India, 74(5), 411-417.

[8] Arishi, Abdulaziz A., et al. "Knowledge, attitude, and perception of robotic-assisted surgery among the general population in Saudi Arabia: a cross-sectional study." Journal of Robotic Surgery 18.1 (2024): 196.
